# Intravenous Methadone for Severe Cancer Pain: A Presentation of 10 Cases

**DOI:** 10.1155/2013/452957

**Published:** 2012-11-28

**Authors:** D. Lossignol, I. Libert, B. Michel, C. Rousseau, M. Obiols-Portis

**Affiliations:** Supportive and Palliative Care Unit, Institut Jules Bordet, Waterloo Boulevard 121, 1000 Brussels, Belgium

## Abstract

*Purpose*. Methadone, a synthetic opioid agonist, is an effective alternative to strong opioids (morphine, hydromorphone, oxycodone, and buprenorphine) and is widely available as an oral formulation. Few data have been published so far on the use of intravenous (i.v.) methadone for the management of severe or refractory cancer pain. *Methods*. We followed 10 consecutives cancer patients with severe pain, treated with IV methadone. All had advanced disease and had already received strong opioids, some in association with ketamine. Pain was assessed at T0, T24 hours, and at the end of the treatment. *Results*. All patients benefited from the switch to IV methadone with a reduction of pain on VAS after 24 hours (median: 4/10; range 0–5) until the end of the treatment (all cases <3/10). The median starting dose was 100 mg/day (range 20–400) and the final dose remained stable with a median of 100 mg/day (range 27–700). The median duration of IV methadone was 11 days (range 2–59). No cardiac toxicity had been observed. *Conclusions*. IV methadone is an effective pain relieving alternative for the treatment of severe cancer pain, especially in refractory pain syndrome. Moreover, we did not observe any toxicity (neurological or cardiac) or any other major side effects and the treatment was overall well tolerated. More extensive comparative studies should be planned.

## 1. Introduction

The use of strong opioids remains the corner stone of pain management in cancer. Methadone, a synthetic opioid agonist, is an effective alternative to strong opioids (morphine, hydromorphone, oxycodone, and buprenorphine) and is widely available for oral formulation. Methadone has no known active metabolite and is part of the strategy of opioid rotation [1–9]. Numerous studies suggest that the equianalgesic depends on the previous opioid treatment but there are few data on the use of intravenous (IV) methadone for the management of severe or refractory cancer pain [5, 6]. Cardiac toxicity is potentially of concern, especially because of the risk of arrhythmia, “torsades de pointes” and sudden death [7, 8]. The use of IV methadone is often suggested but rarely reported [10].

We present our preliminary experience with IV methadone in 10 cancer patients with advanced disease and severe cancer pain already treated with high doses of opioids. 

## 2. Patients and Methods

Ten consecutive patients with advanced cancer (progressive disease) were referred to our 12 beds unit for symptoms management and “intractable pain.” All had previously received various treatments for pain (step I to step III WHO ladder and various coanalgesics) and were not controlled at the time of evaluation despite dose escalation or opioids rotation. They all presented with refractory pain and undesirable side effects from pain treatment and were considered at a palliative stage (no more ongoing or planned chemotherapy, radiation therapy, or surgery). 

### 2.1. Treatment Plan

Our criteria to introduce IV methadone were (1) advanced-severe cancer pain, (2) poor pain control with high doses of step III opioids, (3) side effects from related pain treatment: dizziness, nausea, and myoclonus, (4) a neuropathic pain component, (5) no previous ineffective treatment with (oral) methadone, (6) no cognitive impairment (capability to understand information), and (7) renal dysfunction.

We used a 10 : 1 ratio from morphine to methadone in most cases. For other opioids, we first used the “morphine equivalent daily dose” (MEDD) before switching to methadone: *morphine* : *hydromorphone*: 7.5 : 1*, morphine* : *oxycodone*: 2 : 1, and* morphine (parenteral) : fentanyl (transdermal)*: 80 : 1 [1]. The converting ratio for oral to intravenous methadone was 3 : 1. All patients had an electrocardiogram (ECG) to measure the QT interval before the introduction of IV methadone. A control ECG was planned once a week or according to clinical circumstances. In some cases, ketamine was considered if a breakthrough pain component was present (it must be emphasized that fentanyl citrate is not available in Belgium and there is no reimbursement foreseen). All continuous opioids (not the rescue doses) were discontinued four hours before starting IV methadone. 

### 2.2. Pain Evaluation

Pain was systematically measured with a visual analog scale (VAS), at T0 (before to start IV methadone), T24, and at the end of treatment. Of course, pain was also evaluated every day by nurses and/or the medical team. The primary endpoint was an optimal pain control. Other endpoints were adequate control of side effects, intravenous methadone tolerance, cognitive status, and cardiac toxicity.

## 3. Results

### 3.1. Patient Characteristics

Patient characteristics are shown in Table 1. Most patients were female with gynecological cancer. The median age was of 48 (range 20–64) for women and 49 (range 41–49) for men. Pain was severe in all cases, between 7/10 and 9/10. All patients were already treated with high doses of opioids and two patients already had receive an association of hydromorphone and ketamine (patient 1 and 10). The median MEDD was 2400 mg (range 450 mg–6750 mg). Five patients already received oral methadone (patients 2, 3, 4, 6, and 8). They all had uncontrolled breakthrough pain episodes. Radiation therapy for pain management has been administered earlier in most cases without effective pain relief. All pain syndromes expressed a neuropathic component, essentially due to locoregional involvement, lumbosacral plexopathy, multiple bone metastases or leptomeningeal, and spinal cord involvement. ECG before IV methadone was normal in all cases.

### 3.2. Intravenous Methadone

All patients received IV methadone according to a converting ratio with the MEDD, with a median dose of 100 mg (range 20–400). The median MEDD was calculated as 3000 mg (range, 180–12000), and previous rescue doses (hydromorphone, morphine, and oxycodone) were taken into account when calculating the total dose of methadone. Methadone for perfusion was prepared without preservative by the hospital pharmacist. Median duration of treatment was 11 days (range 2–59). The final dose of methadone was higher in most cases despite a stable median of 100 mg (range 25–700) (see Table 2 for details).

### 3.3. Pain Evaluation

After 24 hours of IV methadone, all patients achieved a better pain control with a median of VAS of 4/10 (range 0/10–5/10). Pain remained under controlled until the end of treatment. Five patients received ketamine in association with methadone. The main reason was a breakthrough pain component (from 2 to 4 episodes a day).

### 3.4. Safety

The main side effect of IV methadone was a transient sedation. ECGs remained normal in all cases. All patients but one died from disease progression.

## 4. Discussion

Our data show that intravenous methadone may be used for the treatment of severe cancer pain with a rapid pain relief, without unbearable side effect. Because of the wide variation in the duration of treatment, we could not demonstrate long-term safety, although some of our patients received IV methadone for more than 20 days without toxicity.

The use of methadone in the so-called “opioids rotation,” is well established for years, essentially because there is no absolute cross-tolerance with the other types of opioids. The question of the equianalgesic dose ratio between methadone, morphine, and other opioids is not completely settled, particularly, in patients tolerant to high doses of opioids; but there is an agreement to consider a ratio from 4 : 1 to 10 : 1 according to previous opioid dosage [1–9]. We adopted the 10 : 1 ratio, because the dosage of opioids was very high in all patients, prior to the switch to methadone. Methadone has no active metabolites, is essentially excreted through the biliary tract, and can thus be used in cancer patients with renal function impairment. These potential advantages have to be weighed against relative risk of QT prolongation and the development of “torsades de pointes” potentially leading to fatal arrhythmias [7, 8]. However, we did not observe such a complication in our patients. Methadone has an antagonist action against N-Methyl-D-Aspartate (NMDA) receptors, which are associated to the tolerance to opioids and also to the development of chronic pain [11]. We had to resort to IV ketamine in 5 patients who presented with paroxystic painful episodes; this suggests that the effect of methadone on the NMDA receptors is only limited. The necessary dose of IV methadone remained stable in most of our patients. It is interesting that in 5 patients who were receiving methadone PO, the IV administration improved dramatically the control of pain, maybe through a better biodisponibility of the medication. Although it is generally accepted that the use of IV methadone requires a close medical monitoring, our results suggest that eventually the potential risks are not encountered very often and that, therefore, this therapeutic approach can be safely recommended for routine management of refractory pain in cancer.

In conclusion, IV methadone is an effective pain relieving alternative for the treatment of severe cancer pain, especially in refractory pain syndromes, at the end of life. Although the number of cases is small in this series, we did not observe any significant toxicity and the treatment was well tolerated with an effective pain relief. As our patients did not present any cognitive impairment during the treatment, it is likely that IV methadone might be an alternative to “terminal sedation,” whichissometimes proposed to control refractory pain. Further studies are necessary to confirm our data and to determine the right time to use IV methadone and its place in routine practice.

## Figures and Tables

**Table 1:** Patient's characteristics.

Patients	Sex	Age	Type of cancer	Pain treatment (mg/day)
1	F	32	Vulvar sarcoma	Hydromorphone 300/ketamine 150 (i.v.)
2	F	48	Lung (SCLC)	Oral methadone 210
3	M	49	Rectum	Oral methadone 180
4	F	20	Ewing sarcoma	Oral methadone 240
5	M	49	Colon	Morphine 900 (i.v.)
6	F	60	Vulvar carcinoma	Oral methadone 240
7	F	64	Vulvar carcinoma	Hydromorphone 50 (i.v.)
8	F	37	Vaginal carcinoma	Oral methadone 45
9	F	55	Uterine carcinoma	Fentanyl 300 (*μ*g/H)
10	M	41	Pelvic angiosarcoma	Hydromorphone 130/ketamine 100

**Table 2:** Intravenous methadone.

Patients	VAS T0∗(/10)	Starting dose (mg/day)	Ketamine (mg/day)	VAS T24∗ (/10)	Final dose (mg/day)	Ketamine (mg/day)	VAS (end of treatment∗ (/10)	Duration (days)
1	8	400	150	4	700	500	0-1	59
2	8	80	/	0	80	/	0–3	2
3	9	200	200	5	400	960	0	24
4	8	100	200	0	100	200	<3	9
5	8	100	/	4	120	/	0	7
6	7	80	/	4	200	/	2	11
7	9	30	/	0	30	/	0	7
8	8	20	50	4	25	50	0	14
9	8	30	/	3	30	/	0–2	10
10	9	100	100	4	90	80	<3	25

^*^On average.
